# Ultrasound combined with serum parathyroid hormone for assessing autologous parathyroid graft viability after endoscopic thyroidectomy: a retrospective study

**DOI:** 10.3389/fendo.2026.1871111

**Published:** 2026-06-29

**Authors:** Qi Zhang, Tingbao Cao, Qiongyu Yang, Zhiheng Yan, Yupeng Zhang, Zhao Jin, Zesheng Wang, Kunpeng Qu

**Affiliations:** 1 Department of General Surgery, Gansu Provincial Center Hospital, Lanzhou, China; 2 Department of Ultrasound, Gansu Provincial Center Hospital, Lanzhou, China

**Keywords:** autologous parathyroid transplantation, endoscopic thyroidectomy, hypoparathyroidism, parathyroid hormone (PTH), ultrasonography

## Abstract

**Objective:**

To investigate the diagnostic value of ultrasonography combined with serum parathyroid hormone (PTH) measurement in assessing the viability of autologous parathyroid grafts following endoscopic radical thyroidectomy for thyroid carcinoma.

**Methods:**

A retrospective analysis was performed on clinical data of 38 patients who underwent endoscopic unilateral radical thyroidectomy combined with autologous parathyroid transplantation at Gansu Provincial Central Hospital from January 2023 to September 2025. During surgery, parathyroid tissue was transplanted into the brachioradialis muscle of the forearm using the homogenate injection method. Postoperatively, PTH concentration ratios in blood samples from both antecubital fossae and ultrasonographic images of the graft site were periodically evaluated to assess graft viability.

**Results:**

Parathyroid hormone concentration on the transplantation side increased persistently after surgery, reaching a peak at 3 months postoperatively, with a significant elevation in the PTH ratio (28.89-fold) compared to the systemic circulation. Thereafter, the ratio gradually declined but consistently remained >1.5-fold. Ultrasonography performed at 3 months postoperatively revealed hypoechoic nodules of approximately 3 mm in diameter at the graft site in some patients, with punctate blood flow signals detected by color Doppler flow imaging (CDFI).

**Conclusion:**

The combination of ultrasonography and serum PTH concentration ratio provides objective imaging and biochemical evidence for parathyroid graft viability following autologous transplantation, facilitating more accurate assessment of functional recovery of the graft.

## 1 Introduction

Hypoparathyroidism is one of the most common complications following thyroidectomy (1). The strategy to prevent its complications is to strategically transplant one inadvertently resected or devascularized parathyroid gland based on the *in situ* preservation of at least one parathyroid gland (2). In 1996, Olson et al. (3) selected the brachioradialis muscle of the forearm as the transplantation site, and a PTH concentration ratio of 1.5 in the antecubital venous blood of both arms was considered indicative of graft viability. However, in that study, based on conventional thyroidectomy, a secondary incision was made to transplant the parathyroid gland into the brachioradialis muscle of the forearm using the particle embedding method (4). Subsequently, to avoid a secondary incision during the same surgery, most domestic and international studies (5, 6) embedded parathyroid particles into the sternocleidomastoid muscle. Nevertheless, this approach precluded postoperative assessment of whether the transplanted parathyroid gland exerted PTH secretory function. Our group, based on endoscopic thyroidectomy, utilized the homogenate injection method (4) to transplant inadvertently resected or devascularized parathyroid glands into the brachioradialis muscle of the forearm. The viability and PTH secretory function of the transplanted gland were evaluated by comparing PTH concentrations in the cephalic veins of both antecubital fossae. This method avoids a secondary incision during surgery while enabling postoperative evaluation of graft viability. However, previous studies using this approach lacked imaging evidence of the transplant site. This article, based on endoscopic unilateral radical thyroidectomy combined with autologous parathyroid transplantation, investigates the postoperative serum PTH concentration ratio in the antecubital fossae of both arms and ultrasonographic findings of the transplanted parathyroid gland at the graft site, aiming to establish a more comprehensive diagnostic approach for assessing graft viability.

## 2 Materials and methods

### 2.1 Study subjects

A retrospective analysis was performed on the clinical data of 38 patients who underwent endoscopic unilateral radical thyroidectomy combined with autologous parathyroid transplantation in the Department of General Surgery, Gansu Provincial Central Hospital from January 2023 to September 2025. Postoperative PTH concentrations in the antecubital fossae of both arms at 3 months after surgery and ultrasonographic images of the transplanted parathyroid gland at the graft site were collected to evaluate the viability of the transplanted parathyroid gland. Inclusion criteria were as follows: (1) patients with a postoperative pathological diagnosis confirmed as papillary thyroid carcinoma; (2) all patients underwent unilateral thyroidectomy with isthmusectomy and concurrent autologous parathyroid transplantation. Exclusion criteria were as follows: (1) patients with incomplete clinical data or failure to complete scheduled postoperative follow-up; (2) patients with a history of previous neck surgery or related treatment; (3) patients with abnormal preoperative serum parathyroid hormone or calcium levels; (4) patients who underwent concurrent lateral neck lymph node dissection; (5) patients with concomitant hepatic or renal insufficiency; (6) patients in whom no parathyroid tissue was identified in the postoperative pathological specimen, despite suspected intraoperative identification, and who therefore did not undergo autologous parathyroid transplantation. Written informed consent was obtained from all participating patients, and this study was approved by the Medical Ethics Committee of Gansu Provincial Central Hospital (Approval No. 2022-195).

### 2.2 Surgical method

**Figure 1:**
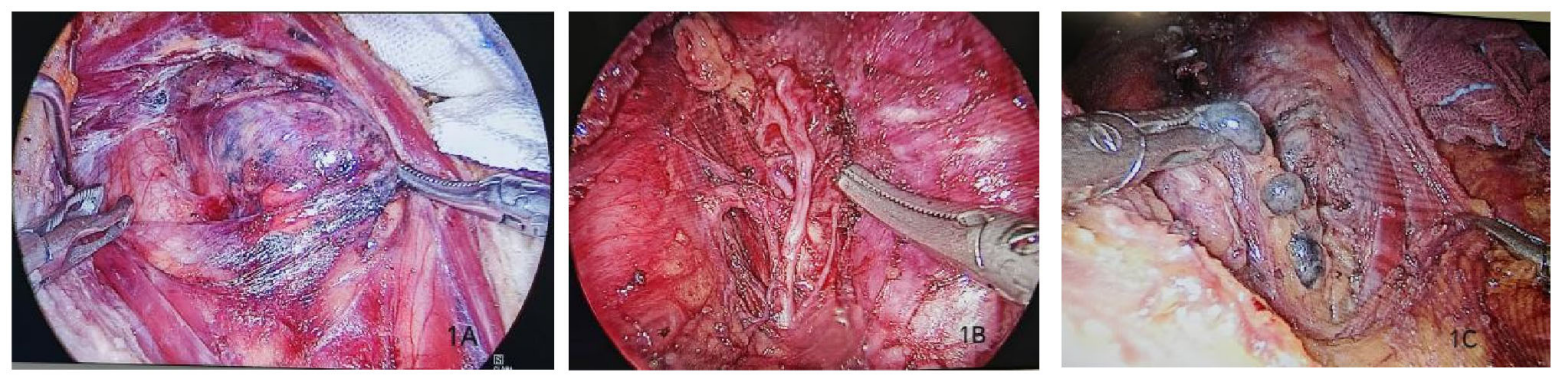
Key anatomical images of endoscopic radical thyroidectomy.

Endoscopic unilateral radical thyroidectomy was performed under general anesthesia. During the procedure, the affected thyroid lobe was first gradually dissected and exposed, followed by percutaneous injection of 0.1 mL of carbon nanoparticle suspension to stain the thyroid tissue and surrounding lymph nodes black. Simultaneously, the negative staining technique was used to identify and preserve the parathyroid glands (**Figure 1A**). Under the premise of meticulous dissection, the affected lobe was completely resected, and important structures such as the recurrent laryngeal nerve, blood vessels, and trachea were carefully dissected and protected (**Figure 1B**). Subsequently, central lymph node dissection was routinely performed (**Figure 1C**). If an inadvertently resected or devascularized suspected parathyroid gland was identified intraoperatively, rapid detection using a PTH immunocolloidal gold test strip was immediately performed for confirmation. Once confirmed as parathyroid tissue, it was promptly placed in 1 mL of normal saline at 4 °C, minced into small fragments using ophthalmic scissors to prepare a cell suspension, and immediately transplanted into the brachioradialis muscle of the non-dominant forearm using the homogenate injection technique. All endoscopic thyroidectomy procedures and subsequent homogenate injections of parathyroid tissue into the brachioradialis muscle were performed by the same senior surgical team to ensure methodological homogeneity.

### 2.3 Outcome measures

The serum PTH concentration ratio in the antecubital fossae of both arms at different postoperative follow-up time points, serum Ca²^+^ concentration, and ultrasonographic images of the transplanted forearm at 3 months postoperatively were documented for all patients. (Note: Normal reference range for serum PTH: 12–88 ng/mL; normal reference range for serum Ca²^+^: 2.11–2.52 mmol/L.).

### 2.4 Postoperative homogenization of confounding factors

To minimize the potential impact of factors known to influence serum parathyroid hormone (PTH) and calcium levels, all patients received a standardized postoperative management protocol. For the first week after surgery, all patients were maintained on a consistent oral calcium supplementation regimen (calcium carbonate 600 mg twice daily) and calcitriol (0.25 μg twice daily). Dietary calcium and vitamin D intake were assessed and standardized through hospital-provided nutritional guidance. Patients with pre-existing or postoperative abnormal renal function (serum creatinine >1.3 mg/dL in females or >1.5 mg/dL in males) were excluded. Vitamin D levels (25-hydroxyvitamin D) were measured at baseline and at each follow-up visit; all included patients maintained 25-hydroxyvitamin D levels >20 ng/mL. No patient received recombinant human PTH or other medications known to interfere with calcium-phosphorus metabolism during the follow-up period. This homogenization protocol ensured that postoperative PTH and calcium measurements reliably reflected graft viability rather than external modulatory factors.

### 2.5 Statistical analysis

Statistical analyses were performed using SPSS version 26.0. Continuous data were expressed as mean ± standard deviation (SD). The normality of data distribution was assessed using the Shapiro–Wilk test. For comparisons of PTH and calcium levels between the transplantation side and non-transplantation side at the same time point, paired t-tests were used for normally distributed data, while Wilcoxon signed-rank tests were applied for non-normally distributed data. A two-sided P value < 0.05 was considered statistically significant.

## 3 Results

### 3.1 Serum PTH and Ca²^+^ concentrations in the antecubital fossae of both arms in 38 patients at different follow-up time points

Preoperatively, the serum PTH ratio between the transplantation side and the non-transplantation side (systemic circulation) was close to 1. At 1 week postoperatively, the PTH ratio exceeded 1.5, reached its peak at 3 months postoperatively, and then gradually decreased but remained above 1.5. Throughout this period, serum calcium concentrations of the patients remained within the normal range (**Table 1**).

**Table 1:** PTH (pg/mL) concentration ratio in the antecubital veins of both arms and serum Ca²^+^ concentration at different time points.

Place	Preop	POD1	POW1	POM1	POM3	POM6	POM12
Transplanted side PTH	45.75 ± 9.68	20.25 ± 9.98	60.88 ± 23.07	288.13 ± 197.03	1210.00 ± 649.61	199.25 ± 99.86	158.25 ± 87.67
Non-transplanted side PTH	45.25 ± 9.22	17.62 ± 9.64	24.50 ± 8.78	32.25 ± 12.57	41.88 ± 11.09	41.38 ± 9.10	43.88 ± 8.48
Ratio (transplantation side PTH/non-transplantation side PTH)	1.01	1.15	2.48	8.93	28.89	4.82	3.61
somatic calcium	2.34 ± 0.06	2.19 ± 0.07	2.20 ± 0.05	2.26 ± 0.04	2.26 ± 0.04	2.31 ± 0.05	2.31 ± 0.07

Preop, Preoperative; POD, Postoperative day; POW, Postoperative week; POM, Postoperative month. The transplantation side represents the PTH derived from the transplanted parathyroid tissue as the secretory source, while the non-transplantation side represents the systemic circulating PTH.

**Figure 2:**
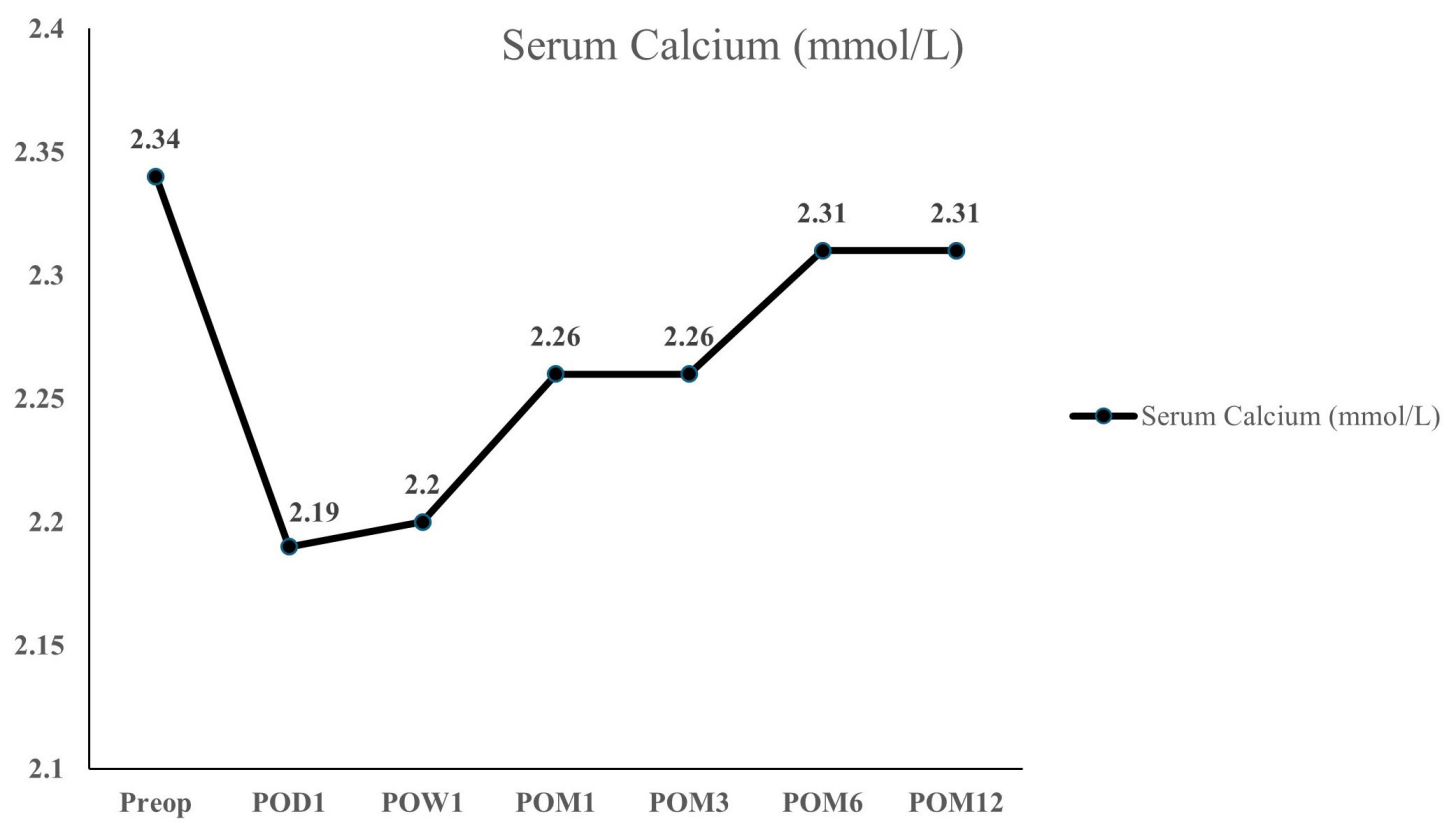
Serum calcium levels over time.

The trend of serum calcium levels over time is illustrated in **Figure 2**. Notably, all mean values remained within the normal range (2.11–2.52 mmol/L) throughout the study period, indicating effective maintenance of calcium homeostasis after autologous parathyroid transplantation (**Figure 2**).

### 3.2 Ultrasonographic images of the transplanted parathyroid gland at different postoperative follow-up periods

**Figure 3:**
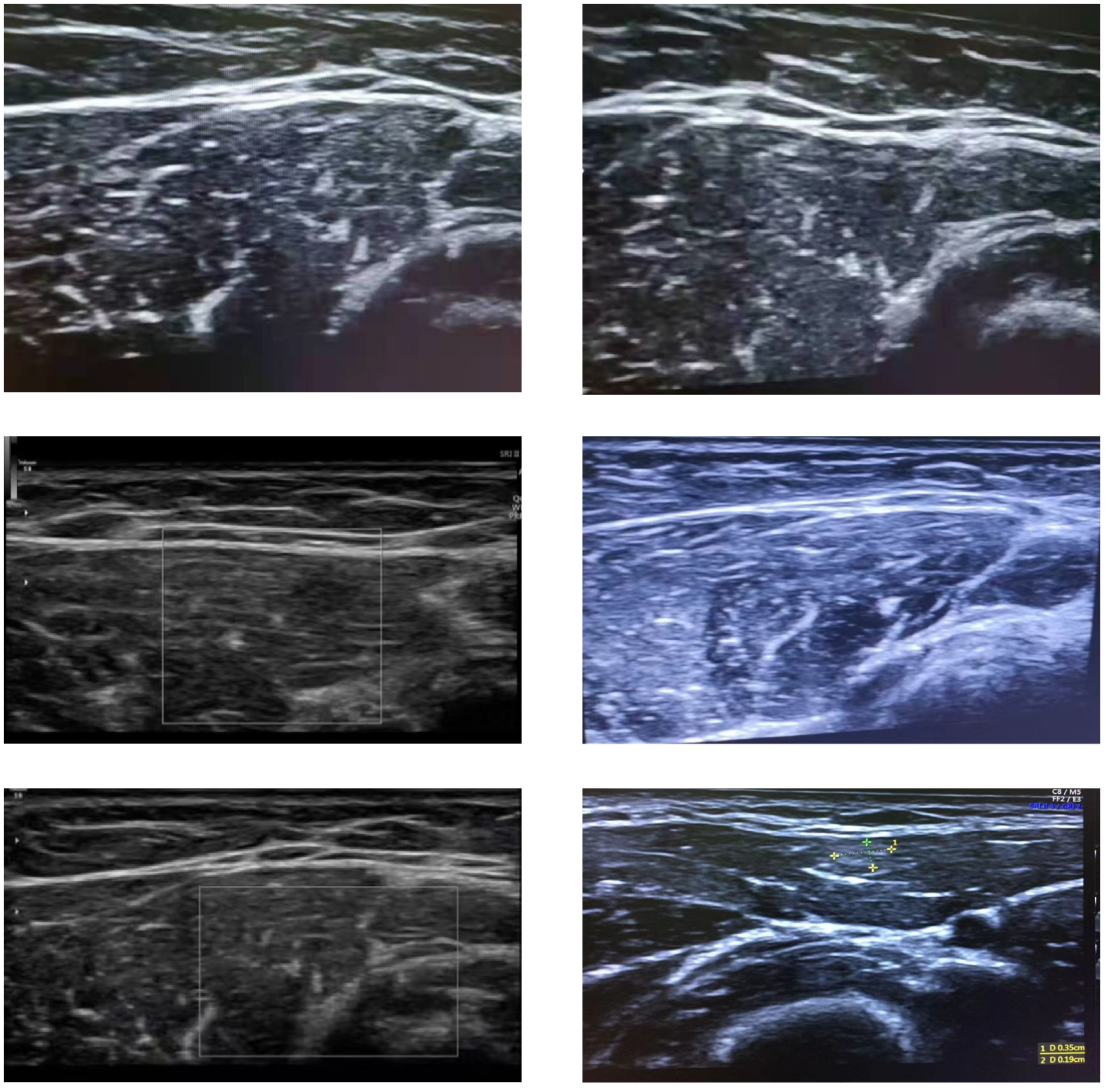
Key anatomical images of endoscopic radical thyroidectomy.

Among the 38 patients followed up, hypoechoic lesions approximately 3 mm in diameter were observed within the brachioradialis muscle on the transplantation side in 34 patients (**Figure 3**), with punctate blood flow signals detected on CDFI. In the remaining 4 patients, no hypoechoic lesion was observed within the brachioradialis muscle on the transplantation side, and no abnormalities were detected on CDFI.

### 3.3 Subgroup analysis: ultrasound-negative patients at any time point and concordance with 3-month PTH ratio

Among the 38 patients, 4 (10.5%) showed no hypoechoic lesion within the brachioradialis muscle on ultrasonography and no detectable blood flow signal on CDFI at any of the postoperative follow-up time points (including 1, 3, 6, and 12 months). For these 4 patients, the PTH ratio (transplantation side/non-transplantation side) was assessed at 3 months postoperatively as the primary functional endpoint. The mean PTH ratio at 3 months was 1.83 ± 0.41 (range: 1.51–2.42), which was above the threshold of 1.5 but significantly lower than that of patients with positive ultrasound findings (28.89 ± 15.32, P < 0.001). Their serum calcium levels remained within the normal range throughout the follow-up period. These findings suggest that the absence of detectable ultrasonographic changes at any time point may be associated with relatively lower functional recovery of the graft, although a PTH ratio >1.5 was still maintained at 3 months.

Among the remaining 34 patients (89.5%), hypoechoic nodules (approximately 3 mm in diameter) with punctate blood flow signals on CDFI were observed within the brachioradialis muscle at 3 months postoperatively or earlier. In this group, 33 patients (97.1%) exhibited a PTH ratio >1.5 at 3 months postoperatively, with a mean ratio of 29.32 ± 16.10. The remaining 1 patient (2.9%) with positive ultrasound findings had a PTH ratio of 1.42 at 3 months, slightly below the predefined cutoff of 1.5, possibly due to incomplete revascularization or partial graft necrosis. These results indicate a high concordance between ultrasonographic evidence of graft survival (observed at 3 months or earlier) and functional evidence based on the 3-month PTH ratio.

### 3.4 Serum phosphorus levels

Serum phosphorus levels were measured at the same time points as PTH and calcium. The normal reference range for serum phosphorus is 0.85–1.51 mmol/L. Preoperatively, the mean serum phosphorus level was 1.16 ± 0.19 mmol/L. On postoperative day 1, the mean phosphorus level increased to 1.48 ± 0.26 mmol/L, then gradually decreased over time: 1.39 ± 0.22 mmol/L at 1 week, 1.30 ± 0.18 mmol/L at 1 month, 1.19 ± 0.15 mmol/L at 3 months, 1.17 ± 0.14 mmol/L at 6 months, and 1.15 ± 0.13 mmol/L at 12 months postoperatively. The initial postoperative elevation in phosphorus levels (still within or near the normal range) corresponded to the transient decline in PTH, while the subsequent normalization of phosphorus levels paralleled the recovery of graft function. These data are presented in **Table 2**.

**Table 2:** Serum phosphorus levels (mmol/L) at different follow-up time points.

Time point	Preop	POD1	POW1	POM1	POM3	POM6	POM12
Serum phosphorus	1.16 ± 0.19	1.48 ± 0.26	1.39 ± 0.22	1.30 ± 0.18	1.19 ± 0.15	1.17 ± 0.14	1.15 ± 0.13

Normal reference range for serum phosphorus: 0.85–1.51 mmol/.L.

### 3.5 Clinical outcomes

Postoperatively, 6 of 38 patients (15.8%) experienced transient mild hypocalcemia symptoms (perioral numbness or fingertip paresthesia) within the first week after surgery. All symptomatic patients received additional calcium supplementation (calcium carbonate 600 mg three times daily) for 3–7 days, and their symptoms resolved without progression to tetany or laryngospasm. No patient developed severe hypocalcemia requiring intravenous calcium infusion. Regarding calcium and calcitriol supplementation, all patients received the standardized protocol (calcium carbonate 600 mg twice daily and calcitriol 0.25 μg twice daily) for the first postoperative week. Beyond the first week, 32 patients (84.2%) required no further supplementation, while 6 patients (15.8%) required continued calcium and/or calcitriol supplementation beyond 1 month postoperatively. By 3 months postoperatively, only 2 patients (5.3%) remained on low-dose calcium supplementation (calcium carbonate 600 mg once daily), and no patients required supplementation beyond 6 months. No adverse events related to hypoparathyroidism or hypercalcemia were observed during the follow-up period.

## 4 Discussion

The parathyroid gland is an important endocrine organ in the human body, regulating calcium and phosphorus metabolism through the secretion of parathyroid hormone (PTH) (7). Normal parathyroid glands are located on the posterior walls of both sides of the thyroid gland, with one superior and one inferior gland on each side. Due to their high fat content, parathyroid glands typically appear on ultrasonography as elliptical, slightly hyperechoic nodules with well-defined borders and homogeneous internal echogenicity, and blood flow signals may be detected within some glands (8, 9). In general, the bilateral inferior parathyroid glands are more readily visualized on ultrasound, being adjacent to or slightly separated from the lower pole of the thyroid gland, which differs from the surgical finding that the superior parathyroid glands are more easily identified. During endoscopic thyroidectomy, thermal injury from instruments, devascularization, or inadvertent resection of the parathyroid glands leads to a reduction in functional parathyroid parenchyma, resulting in postoperative hypoparathyroidism, which increases both the disease burden and economic burden on patients (10). From an anatomical perspective, the superior parathyroid glands have a relatively fixed position and are more likely to be preserved *in situ*, whereas the inferior parathyroid glands exhibit greater anatomical variability and carry a higher risk of intraoperative injury (11, 12). Currently, pharmacotherapy for hypoparathyroidism is limited by the lack of a definitive hormone replacement therapy. Recombinant human parathyroid hormone can replace calcium and vitamin D while improving the abnormal bone remodeling status in patients with hypoparathyroidism (13); however, studies have found that this class of drugs carries a risk of osteosarcoma, which limits their long-term use (14). Therefore, Teshima (15) proposed that preservation of the superior parathyroid glands *in situ* combined with selective transplantation of the inferior parathyroid glands is an effective treatment modality for preserving parathyroid function in cases of inadvertent resection or devascularization injury.

Intraoperative preservation of well-vascularized and functionally intact parathyroid glands *in situ* is more conducive to postoperative recovery of parathyroid function (16). However, due to anatomical variations in the location of the parathyroid glands, a certain risk of intraoperative injury is inevitable. Therefore, some scholars advocate autologous parathyroid transplantation as a feasible strategy to preserve parathyroid function (17). Nevertheless, whether this procedure should be performed routinely remains controversial. Some studies support selective autologous parathyroid transplantation, suggesting that this approach helps reduce the incidence of permanent postoperative hypoparathyroidism (18); other opinions argue that the transplantation procedure itself may increase the risk of transient hypoparathyroidism and does not effectively reduce the occurrence of permanent hypoparathyroidism, thus favoring intraoperative *in situ* preservation of the parathyroid glands (19). Some studies have even suggested that autologous transplantation may paradoxically increase the probability of permanent hypoparathyroidism (20). These discrepancies in conclusions primarily stem from the fact that not all transplanted parathyroid glands survive and resume secretory function, and their survival requires a process of revascularization. Therefore, relying solely on autologous parathyroid transplantation to prevent postoperative hypoparathyroidism is not advisable. Based on the above considerations, our research team adopted a selective autologous parathyroid transplantation strategy during endoscopic thyroidectomy: the inadvertently resected or devascularized inferior parathyroid gland is transplanted into the brachioradialis muscle of the non-dominant forearm, while the superior parathyroid gland is preserved *in situ* whenever possible. This approach aims to minimize the risk of permanent hypoparathyroidism. Postoperatively, the functional recovery of the transplanted gland is assessed based on the PTH concentration ratio in the cephalic veins of both antecubital fossae, and the imaging findings of the graft site are evaluated using ultrasonography to comprehensively determine parathyroid graft viability. Following homogenate injection, normal parathyroid tissue is difficult to visualize on ultrasound because its diameter is smaller than the minimum resolution of ultrasound, and there is no significant acoustic impedance interface between the parathyroid tissue and the muscle. Once the parathyroid graft survives, its volume gradually increases within the muscle bundles, revascularization is established, and the viable parathyroid graft can be identified using high-frequency ultrasound. Ultrasonographically, it appears as a hypoechoic nodule approximately 3 mm in diameter, with a clear boundary from the surrounding muscle bundles and punctate blood flow signals detectable on CDFI. Combined with the PTH concentration ratio in the bilateral antecubital fossae, the viability of the transplanted parathyroid gland can be confirmed. Conversely, the absence of these findings indicates graft failure. In the present cases, ultrasonographic visualization of the graft site further substantiated the viability of the transplanted parathyroid glands.

The identification and preservation of the parathyroid glands are fundamental to the safety of thyroid surgery. Intraoperatively, every effort should be made to identify all parathyroid glands, employing meticulous dissection techniques with maneuvers performed close to the thyroid capsule to maintain the integrity of the parathyroid blood supply as much as possible. When a parathyroid gland is found to be damaged or inadvertently resected during surgery, autologous parathyroid transplantation should be performed immediately. Currently, the assessment of intraoperative parathyroid dysfunction primarily relies on subjective intrajudgment. Under ischemic conditions, the parathyroid gland typically does not cause an obvious color change in the thyroid gland; however, during venous congestion, its color may change from orange to dark brown. In contrast, the impact of ischemia on parathyroid function is often more significant. Therefore, relying solely on color changes to assess glandular functional status is inadequately standardized and cannot accurately distinguish whether the gland has only transient functional impairment or has progressed to permanent loss of function. Given that the half-life of PTH is 4–5 minutes, intraoperative real-time PTH monitoring is recommended (21). When PTH levels fall below the normal range, intraoperative parathyroid injury should be suspected, and the injured gland should be promptly identified based on concomitant changes in parathyroid color. Alternatively, indocyanine green fluorescence angiography (22) can be used to evaluate parathyroid blood supply, providing a more objective assessment of parathyroid function and aiding in the decision of whether to perform autologous parathyroid transplantation. Postoperatively, we routinely administer calcium supplementation and calcitriol. Serum PTH and calcium levels are measured within 24 hours after surgery. If both values fall within the normal range, calcium supplementation is discontinued; otherwise, the calcium dosage is adjusted according to the laboratory results until the transplanted parathyroid gland survives and exerts its PTH secretory function.

Evidence of graft viability is primarily based on the concentration of PTH in the systemic circulation. However, there are two sources of PTH in the systemic circulation: the parathyroid gland preserved *in situ* and the transplanted parathyroid gland. In the present study, the PTH concentration on the transplantation side increased over time, reaching its peak secretory status in the antecubital venous blood on the transplantation side at 3 months postoperatively, with a ratio of 28.89-fold compared to the systemic concentration. Thereafter, the PTH secretory function of the transplanted gland gradually declined but consistently remained greater than 1.5-fold relative to the systemic circulation, whereas systemic PTH and Ca²^+^ concentrations remained within the normal range throughout. The observed 28.89-fold elevation in PTH concentration on the transplantation side at 3 months postoperatively warrants further explanation. A plausible mechanism is the localized, undiluted secretion of PTH from the surviving graft directly into the venous drainage of the brachioradialis muscle, where the antecubital vein sampling site is located. Unlike the systemic circulation, which receives PTH from both the in-situ preserved glands and the graft, the antecubital vein on the transplantation side primarily reflects graft-derived PTH. Additionally, the early postoperative microenvironment within the muscle may promote neovascularization and transiently enhance graft function. However, the exact mechanism remains incompletely defined, and the lack of direct experimental validation is acknowledged as a study limitation.In 89% of patients, ultrasonography of the transplanted forearm revealed uniform hypoechoic nodules within the brachioradialis muscle, with blood flow signals detectable on CDFI. Our team’s research demonstrates that serum PTH levels show a continuous increasing trend with prolonged postoperative recovery time, generally stabilizing between 3 and 6 months after surgery. During this process, the parathyroid gland preserved *in situ* gradually recovers its PTH secretory function. Concurrently, angiogenesis occurs between the transplanted parathyroid gland and surrounding tissues, leading to revascularization and ultimately achieving an optimal state of PTH secretion (23). Two possibilities exist in this context. First, if the PTH secretory function of the parathyroid gland preserved *in situ* is insufficient to meet the body’s requirements, the transplanted parathyroid gland will maintain long-term PTH secretory function. Second, if the parathyroid gland preserved *in situ* survives and recovers normal PTH secretory function sufficient to meet the body’s demands, this may negatively feedback and inhibit the PTH secretory capacity of the transplanted parathyroid gland. Whether the transplanted gland subsequently loses its PTH secretory function or remains in a quiescent state requires further follow-up to confirm.

In addition to PTH and calcium, serum phosphorus is another key mineral regulated by parathyroid hormone. PTH promotes renal phosphate excretion by inhibiting phosphate reabsorption in the proximal tubules, thereby lowering serum phosphorus levels. In the setting of post-thyroidectomy hypoparathyroidism, decreased PTH secretion leads to reduced renal phosphate excretion, resulting in elevated serum phosphorus levels. In the present study, we observed a transient postoperative elevation in serum phosphorus (1.48 ± 0.26 mmol/L on POD1), which remained within or near the normal range (0.85–1.51 mmol/L) and gradually normalized to 1.19 ± 0.15 mmol/L at 3 months and 1.15 ± 0.13 mmol/L at 12 months, paralleling the recovery of PTH secretion from the graft. The inverse relationship between PTH and phosphorus levels further supports the functional recovery of the transplanted parathyroid tissue. Therefore, monitoring serum phosphorus, in addition to calcium and PTH, provides a more comprehensive biochemical profile for assessing graft viability and patient recovery. We suggest that future studies incorporate phosphorus measurement as an adjunctive marker of parathyroid function.

The diagnostic value of ultrasound assessment for the transplanted parathyroid gland is exceedingly important. This non-invasive, cost-effective, and widely available imaging modality provides multifaceted information that aids physicians in comprehensively understanding the patient’s condition and postoperative status. First, ultrasound helps determine the exact location of the transplanted parathyroid gland, which is crucial for surgical planning and postoperative monitoring. Second, it provides structural information about the transplanted parathyroid gland, including its size, shape, and internal architecture, facilitating early detection of potential abnormalities such as hyperplasia or neoplastic lesions. Furthermore, ultrasound can assess the vascularity of the transplanted parathyroid gland. For patients undergoing parathyroid transplantation, ultrasound assessment can be used for postoperative monitoring of the graft status, helping to determine whether the transplantation has been successful and whether adjustments to the treatment plan are necessary. Additionally, ultrasound involves no radiation exposure and is relatively safe for patients, particularly in cases requiring repeated examinations. In summary, ultrasound assessment provides important diagnostic information for the management of patients with transplanted parathyroid glands, assisting physicians in diagnosing problems, monitoring postoperative status, and guiding treatment decisions. Although there may be certain limitations in some circumstances, it plays a key role in clinical practice as a rapid and reliable tool. Final diagnostic and treatment decisions should be based on comprehensive consideration of the ultrasound assessment findings, clinical symptoms, and other relevant information.

Several limitations of this study should be acknowledged. First, this was a retrospective, single-center study with a relatively small sample size (n=38), which limits the generalizability of our findings. Second, the follow-up period was limited to 12 months postoperatively; longer follow-up is needed to determine whether the transplanted gland maintains function over time or undergoes suppression after recovery of in-situ glands. Third, although we implemented a standardized postoperative protocol to homogenize potential confounding factors—including uniform calcium and calcitriol supplementation for the first postoperative week, nutritional guidance, renal function screening, and vitamin D level maintenance (>20 ng/mL)—other unmeasured factors cannot be entirely ruled out. Fourth, a control group without transplantation was not included because all enrolled patients had intraoperatively confirmed devascularized or inadvertently resected parathyroid tissue, making autotransplantation clinically necessary and a no-transplantation control neither ethically nor practically feasible. Fifth, the 3-mm hypoechoic nodules detected by ultrasonography were not pathologically or cytologically confirmed to be viable parathyroid tissue, and the possibility of other soft-tissue lesions cannot be completely excluded. Sixth, the observed 28.89-fold elevation in PTH concentration on the transplantation side, while plausible due to localized undiluted secretion, lacks direct experimental validation. Seventh, the absence of ultrasonographic evidence in four patients (despite PTH ratios >1.5 at 3 months) suggests that ultrasound alone may not be sufficiently sensitive to detect all viable grafts, while one patient with positive ultrasound findings had a PTH ratio below 1.5 at 3 months, indicating that imaging and functional assessments do not always fully align. Taken together, these limitations—including the small sample size, short follow-up duration, retrospective and single-center design, lack of a control group, absence of pathological confirmation of ultrasound lesions, and incomplete adjustment for confounding factors—further constrain the strength of our conclusions. Therefore, our findings should be considered preliminary, and prospective, multicenter studies with larger cohorts, longer follow-up, pathological confirmation of ultrasound findings, and inclusion of a control group (where ethically feasible) are warranted to validate the combined role of ultrasound and PTH assessment in evaluating autologous parathyroid graft viability.

Autologous parathyroid transplantation is an effective measure to prevent hypoparathyroidism following endoscopic thyroidectomy. However, most current studies rely solely on serum PTH levels to assess graft viability, with no reports on ultrasonographic imaging of the transplanted parathyroid gland at the graft site. Based on serum PTH concentration, the addition of ultrasonographic examination to observe the location, size, number, morphology, and blood perfusion of the transplanted parathyroid tissue in the patient’s forearm will provide more robust imaging support for evaluating graft functional recovery.

## Data Availability

The original contributions presented in the study are included in the article/supplementary material. Further inquiries can be directed to the corresponding author.
